# Patient self-management of warfarin therapy – a long-term follow up study

**DOI:** 10.1186/s12959-025-00694-z

**Published:** 2025-02-10

**Authors:** Erland Hegardt Hall, Marit Holm Sølsnes, Sverre Sandberg, Una Ørvim Sølvik

**Affiliations:** 1 Department of Laboratory Medicine, Nordland Hospital in Bodø, Bodø, Norway; 2 Department of Cardiology, Nordland Hospital in Bodø, Bodø, Norway; 3 Norwegian Organization of Quality Improvement of Laboratory Examinations (Noklus), Haraldsplass Deaconess Hospital, Bergen, Norway; 4 Department of Medical Biochemistry and Pharmacology, Haukeland University Hospital, Bergen, Norway; 5 Department of Global Public Health and Primary Care, Faculty of Medicine, University of Bergen, Bergen, Norway

**Keywords:** Patient self-management, Conventional treatment, Anticoagulation treatment, Complications, TTR, INR variation, Extreme INR values

## Abstract

**Background:**

Patient self-management (PSM) of anticoagulant treatment with vitamin K antagonist (VKA) has emerged as an effective approach for maintaining the international normalized ratio (INR) within the therapeutic range. The objective of this quality assurance project, conducted in clinical practice, was to evaluate the long-term effectiveness and safety of anticoagulant treatment with warfarin during PSM compared to conventional treatment administered by general practitioners (GPs).

**Methods:**

This cohort study, using a retrospective and prospective design, included 400 patients who underwent PSM training for a 21-week period between 2011 and 2020. Clinical data extracted from the patient journal systems included hospitalization due to severe clinical complications. The primary outcome was any difference in the yearly risk of hospitalization between the conventional and PSM periods. Secondary outcomes included variations in time within the therapeutic range (TTR), INR fluctuations, and incidence of extreme INR values.

**Results:**

The median treatment duration was 2.45 years (25th—75th percentile 0.80, 7.35) for the conventional period and 4.99 years (25th—75th percentile 2.41, 7.43) for the PSM period. The annual risk for hospitalization due to severe bleeding was 1.25% during PSM compared to 1.69% during conventional treatment (*p* = 0.885). The yearly risk for hospitalization due to thrombosis was 0.67% during PSM versus 1.48% during conventional treatment (*p* = 0.256), and the annual risk for hospitalization due to spontaneous bleeding, thrombosis, or thromboemboli was 1.12% versus 2.76% (*p* = 0.112). Median TTR (25th—75th percentile) increased from 71.6% (60.0, 82.7) to 78.6% (67.9, 91.7) (*p* < 0.001), while INR variance decreased from 21.0% to 16.5% (*p* < 0.001). The proportion of extreme subtherapeutic INR values (≤ 2.0 (≤ 1.5 for patients with mechanical ON-X aortic valve prostheses)) decreased from 14.0% to 5.0% (*p* < 0.001) during PSM, whereas the proportion of high-level INR (≥ 5.0) remained unchanged (0.6%).

**Conclusions:**

The long-term evaluation of PSM of warfarin treatment in clinical practice suggests that PSM for suitable patients selected by GPs is as safe as conventional GP treatment.

## Introduction

Patients with mechanical heart valve prostheses, deep vein thrombosis and pulmonary embolism related to thrombophilia disorders, cardiac aneurysms with thrombi, or instances of treatment failure with DOACs (direct oral anticoagulants) are managed using a vitamin K antagonist (VKA) such as warfarin. Warfarin, a synthetic coumarin derivative, prevents blood clot formation but concurrently poses an elevated risk of bleeding and potential therapy failure due to drug interactions, dietary changes, and lifestyle adjustments. In Norway, conventional VKA therapy is administered by general practitioners (GPs), involving International Normalized Ratio (INR) monitoring and warfarin dosing typically scheduled at 2–4-week intervals. Consequently, prolonged periods of INR levels outside the therapeutic range and increased risks of bleeding or thrombosis may go undetected.

Patient self-management (PSM) of VKA therapy represents an empowering concept wherein trained patients undertake the responsibility of monitoring their INR levels and adjusting warfarin dosages once a week in a home setting. Meta-analyses of randomized clinical trials (RCTs) have consistently demonstrated that PSM of VKA therapy significantly reduces the risk of complications when compared to conventional management [1–5]. Long-term studies spanning 3–7 years in European countries have further shown a notable decrease in mortality rates with PSM compared to conventional treatment [6, 7]. Additionally, international investigations conducted within clinical settings have underscored the safety of PSM in VKA therapy [6–13]. Nonetheless, few clinical practice studies include a -matched control group for comparison [13]. Both national and international research have consistently highlighted the benefits of PSM in VKA therapy, including longer time in the therapeutic target range (TTR), reduced INR variability, and fewer instances of extreme INR values compared to conventional treatment [1, 14–20].

In a pilot project at Nordland Hospital in Bodø, Norway, patients with mechanical heart valves underwent training in PSM back in 2008. This training program, developed in accordance with international guidelines [21], was further refined by The Norwegian Organization for Quality Improvement of Laboratory Examinations (Noklus) based in Bergen, Norway, as previously described [19]. Collaboratively, Noklus and various hospitals across Norway have successfully trained approximately 2000 patients in PSM. Specifically, between 2011 and 2020, Nordland Hospital in Bodø has trained 400 individuals. A prior study conducted a comprehensive evaluation of Noklus' PSM training program over a two-year follow-up period, revealing significant benefits including a higher TTR, reduced variability in INR levels, fewer instances of extreme INR values, and an overall improvement in quality of life compared to conventional treatment [19].

This quality assurance project, conducted within real-life clinical practice, aimed to evaluate the long-term effectiveness and safety of anticoagulant treatment during PSM compared to prior conventional treatment administered by GPs. We also aimed to examine any differences in TTR, variation in INR and extreme INR-values during PSM compared with conventional treatment.

## Methods

### Study design

The study was longitudinal with a retrospective and prospective design where the same cohort were followed during conventional treatment by GPs and after training period in patient self-management (PSM).

### Training in self-management

The training in self-management spans over 21 weeks and is structured into three comprehensive course days. During these sessions, learning about the theoretical background and principles for warfarin dosing and INR dynamics was highlighted. The participants receive hands-on instruction and individualized training in utilizing the handheld INR point-of-care instrument, alongside guidance on adjusting warfarin doses when INR levels deviate from the target range. To progress to self-management, participants must successfully pass a written assessment, demonstrating both comprehension of the course material and proficiency in dose adjustment protocols. Following patient approval for self-management, the patients are recommended to continue to measure INR once a week, and it is advised that GPs conduct checks of the INR values stored in the INR point-of-care instrument during patient visits to the GP office for parallel measurement 2–3 times a year. Percentage deviation must be < 20% for a single sample and average deviation < 10% for repeated parallel measurements. The 21-week long training program in self-management of warfarin therapy has previously been described in more detail [19].

### Study population

All 400 patients trained in self-management of warfarin treatment at Nordland Hospital in Bodø between 2011 and 2020 were notified about the study via a letter. As the study was categorized as a quality assurance study, obtaining informed consent for the collection of clinical events relating to anticoagulant treatment from their hospital records was not required. The recruitment process for patients requiring long-term or life-long warfarin therapy for training in PSM was conducted at GP offices, the cardiac outpatient clinic, and the postoperative care and rehabilitation unit at Nordland Hospital in Bodø, Norway. Each patient's suitability for warfarin self-management was assessed by their physician, considering factors such as physical health, mental suitability, motivation, and anticipated level of compliance with the PSM regimen. Patients with any indications for starting warfarin therapy were included in the study.

### Data collection

Between September 2020 and February 2021, data retrieved from patient journals via DIPS, an e-health provider for specialist healthcare services in Norway, comprised documentation of diagnostic and intervention procedures undertaken to address severe clinical complications. These complications, categorized as severe adverse events (SAEs), encompassed instances of hospitalization due to either bleeding or thrombosis, detailing the severity and causes of mortality since the initiation of warfarin treatment. Bleeding events were classified in accordance with the criteria outlined by the International Society on Thrombosis and Haemostasis [22]. Major bleeding events were defined as necessitating hospitalization, blood transfusion, fall in hemoglobin level of > 2.0 g/dL, manifested intracranially, or resulted in death. Thromboembolic events were defined by the presence of arterial or venous thrombi, visualized by magnetic resonance imaging (MRI), computed tomography (CT) scans, X-rays, or diagnostic ultrasound procedures. Additionally, admissions involving interventions to secure hemostasis and necessitating observation were categorized as clinically relevant non-major (CRNM) incidents.

Patients were asked to complete a questionnaire and provide the last 25 INR values, along with corresponding dates for the PSM period. INR values and associated dates, recommended therapeutic range for the preceding 6–12 months with conventional treatment period prior to self-management training, had already been collected from INR cards or obtained from the GP during patients' enrollment in the training program.

### Statistical analysis

The primary outcome of this study was to assess the difference in the yearly risk of severe clinical complications (SAEs) demanding hospitalization during PSM compared to conventional treatment. The training period for self-management was excluded from analysis to evaluate outcomes during periods without strict control. Total patient-years and the number of patients experiencing complications were utilized to calculate the yearly risk of bleeding and thrombosis requiring hospital assessment and treatment. Secondary outcome measures were the difference in TTR, variation in INR and extreme INR values (INR values ≤ 2.0 (≤ 1.5 for patients with mechanical ON-X aortic valve prostheses) and INR values ≥ 5.0) for both patients with normal and high intensity warfarin treatment during PSM compared with conventional treatment. TTR, defined as the number of patient days with INR values in the therapeutic range divided by the total number of patient days, was calculated as described by Rosendaal and colleagues [23]. In this method, it is assumed that any given change in INR between two measurements is linear. For each INR measurement, the number of days within therapeutic range since the last test was calculated and divided by the number of days since the last test. Finally, the sum of total number of days within therapeutic range was divided by the total number of days and multiplied by 100. INR variance was calculated as coefficient of variation (CV) INR for all patients. TTR and INR variance are calculated based on INR-values from the last 6–12 month in the conventional treatment period. For the PSM period the last 25 INR-values were used for these calculations.

The McNemar test was used to compare the difference in yearly risk of hospitalization due to complications such as bleedings and thrombosis between the conventional management and PSM periods. A control analysis was executed employing General Estimated Equation (GEE). The difference in TTR and INR-variance between the two periods (conventional management and after training in PSM) was normally distributed, and a paired t-test was used to test for differences. The Wilcoxon signed-rank test was applied to evaluate the differences in the proportions of extreme INR values.

To discover any differences in characteristics between the group that provided complete documentation of INR results during the self-management period (“Patients that provided INR-results”), and the entire cohort (“All patients”) (Table 1), the Chi-square test was utilized for categorical variables, and the Mann–Whitney U test for continuous variables due to their non-normal distribution.

**Table 1:** Patient characteristics at baseline before training in self-management of warfarin treatment

	**All patients**	**Patients that provided INR-results**	*p*-value**
Number (N)	400	285	
Gender			
Male, n (%)	290 (72.5)	214 (75.1)	0.449
Female, n (%)	110 (27.5)	71 (24.9)	
Age, median (25th—7th percentiles), years	58.3 (48.7, 64.7)	59.3 (50.0, 65.4)	0.219
Indication for warfarin therapy, n (%)^a^			
Artificial heart valve	126 (32)	105 (37)	0.145
Recurrent DVT/pulmonary embolism, thrombofili	69 (17)	36 (13)	0.098
Myocardial infarction with aneurysm and/or thrombosis	50 (13)	38 (13)	0.748
Recurrent DVT or pulmonary embolism, unknown cause	41 (10)	22 (7.7)	0.259
Atrial fibrillation with or without complication	76 (19)	58 (20)	0.660
Embolic stroke /cerebral infarction	38 (9.5)	28 (9.8)	0.887
Deep venous thrombosis, atypical location	27 (6.8)	19 (6.7)	0.966
Systemic arterial embolic event	15 (3.8)	9 (3.1)	0.678
Thrombophilia disease	87 (22)	53 (19)	0.313
Antiphospholipid syndrome (APS)	40 (10)	21 (7.4)	0.233
Factor V mutation (Leiden)	30 (7.5)	20 (7.0)	0.811
Protein C/S deficiency	18 (4.5)	10 (3.5)	0.518
Anti-thrombin deficiency	2 (0.50)	2 (0.70)	0.733
Warfarin treatment before training (conventional treatment), median (25th—75th percentiles), years	2.4 (0.8, 7.3)	2.6 (0.8, 7.3)	0.816
INR target value, n (%)			
Low (< 2.5)	20 (5.0)	15 (5.3)	0.877
Normal (2.5)	249 (62)	160 (56)	0.094
Medium high (2.6–2.9)	19 (4.8)	19 (6.7)	0.280
Very high (≥ 3.0)	112 (28)	91 (32)	0.237

**Chi-square test for categorial variables and Mann-Whitney U test for continues variables

^a^Some patients had more than one indication for warfarin treatment

Continuous variables were presented as median and 25th-75th percentile or mean and standard deviation (SD). Statistical analyses were performed using Microsoft Excel, SPSS (27.0.1.0, 28, and 29), and socscistatistics.com.

## Results

**Fig. 1:**
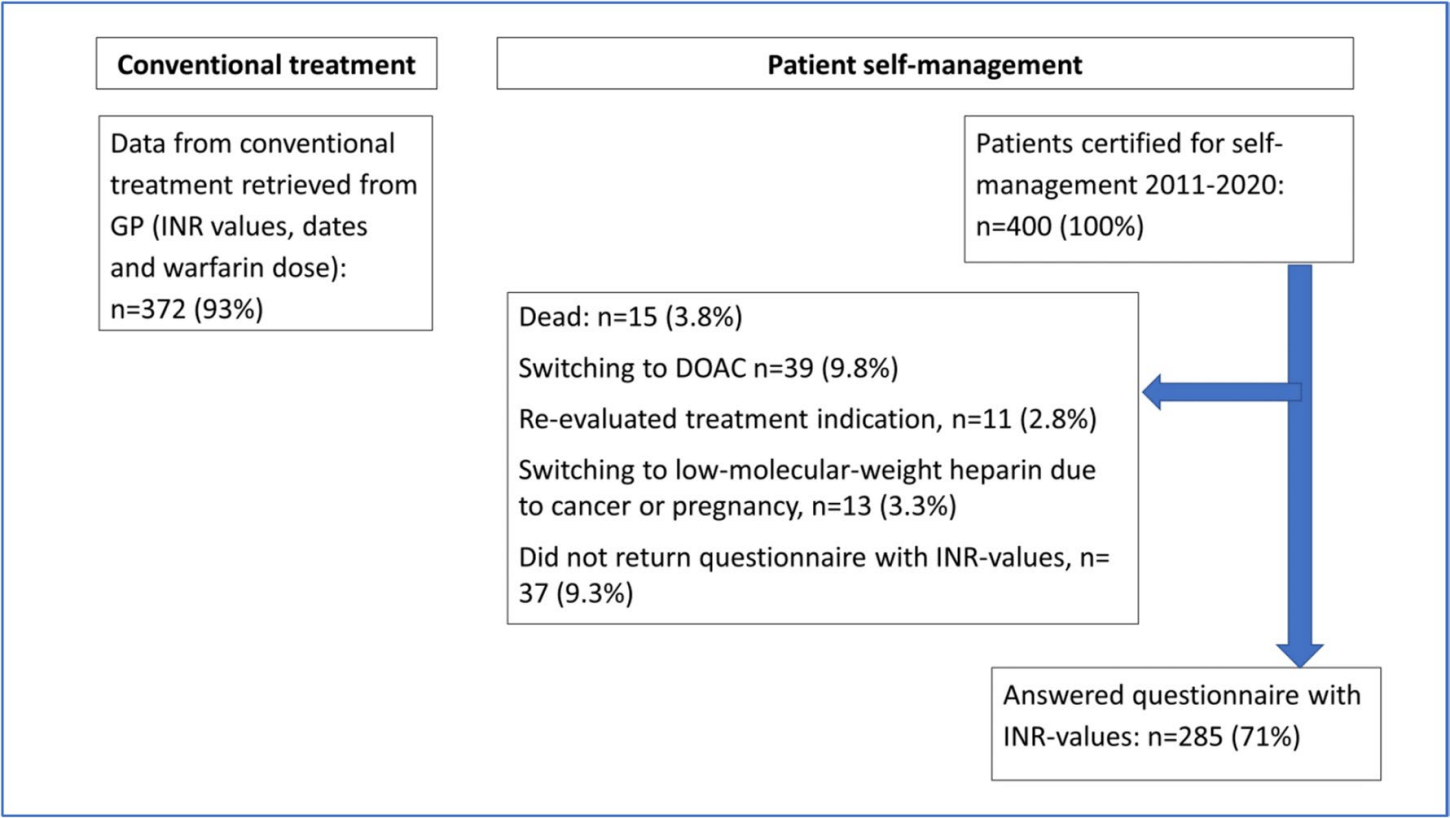
Flow chart showing the number of patients that provided INR-results, forming the basis for the secondary outcome measures, in the conventional treatment period and the patient self-management period. GP = general practitioner, DOAC = Direct Oral Anticoagulant

The baseline characteristics of the 400 patients for whom we have information regarding complications necessitating hospital admission (primary outcome measures), along with indications for warfarin therapy and INR target values are presented as "All patients” in Table 1. At the time of data collection, 322 patients (80.5%) still performed PSM, with 235 of these patients providing the requested information regarding their INR values. Following reminders, an additional 50 out of the 87 initially missing patients submitted the questionnaire. Consequently, a total of 285 patients (71%) responded to the questionnaire and provided the requested INR information which forms the basis for the secondary outcome measures (for details regarding dropouts, see flowchart in Fig. 1). The baseline characteristics including indications for warfarin therapy and INR target values of this subset of patients are presented as "Patients that provided INR-results” in Table 1. Notably, 66 of these patients were also participants in a previous study [19]. However, this previous study had a shorter follow-up duration (maximum two years) and comprehensive details from patients' medical records regarding complications was not available. The median duration of conventional treatment was 2.45 years (25th-75th percentile: 0.80–7.35), accounting for 1958 patient years. The median duration of the PSM period was 4.99 years (25th-75th percentile: 2.41–7.43), encompassing 2242 patient years. In total 13 patients (3.25%) were treated with warfarin for less than 3 months before training in PSM.

Notably, there was a male predominance, and the median ages were 58 and 59 years, respectively, for the two groups. There were no differences between the "All patients" group and the "Patients that provided INR-results" group concerning gender, age, indications for warfarin therapy, and INR target values.

### Risk of complications—Primary outcome measures

There was a trend toward lower annual risk for hospitalization due to severe clinical complications during PSM compared with conventional treatment by GP without reaching statistic significant (Table 2). The GEE-analysis confirmed that there was no difference in severe bleeding, thrombosis or spontaneous bleeding, thrombosis or thromboemboli (Table 2). For the 13 patients that were treated with warfarin for less than 3 months before training in PSM, there were no severe clinical complications in this period.

**Table 2:** Hospitalization due to bleeding or thromboembolic events during conventional treatment and patients’ self-management

Complication	Conventional treatment (*N* = 400, 100%)			Self-management (*N* = 400, 100%)			Mc Nemar	GEE-analysis:			
	(median 2.45 years (25th—75th percentile 0.80, 7.35), 1958 patient years)			(median 4.99 years (25th—75th percentile 2.41, 7.43), 2242 patient years)							
	Total number	Number of patients	Yearly risk, %	Total number	Number of patients	Yearly risk, %	*p*-value	RR (95% CI)	*p*-value	OR (95% CI)	*p*-value
Major bleeding^a^	33	26	1.69	33	28	1.25	0.885	1.08 (0.651, 1.78)	0.773	1.08 (0.631, 1.86)	0.773
Tromboses incl. TIA	29	23	1.48	15	15	0.67	0.256	0.652 (0.340, 1.25)	0.198	0.639 (0.323, 1.26)	0.197
Spontaneous bleeding, thrombosis or thromboemboli	54	38	2.76	27	25	1.12	0.112	0.658 (0.407, 1.06)	0.087	0.635 (0.378, 1.07)	0.087

*RR* relative risk, *OR* odds ratio, *GEE* General Estimated Equation

^a^ISTH-definisjon (International Society on Thrombosis and Haemostasis) [22]

### Time in therapeutic range, INR variation and extreme INR-values—Secondary outcome measures

Median TTR increased from 71.6 (25th-75th percentile 60.0%, 82.7%) during conventional treatment to 78.6% (25th-75th percentile 67.9%, 91.9%) in the PSM period (*p* < 0.001) (Table 3). For the 13 patients that were treated with warfarin for less than 3 months before training in PSM the median TTR increased from 67.8% (25th-75th percentile 44.0%, 88.9%) during conventional treatment to 76.3% (25th-75th percentile 73.4%, 92.0%) in the PSM period. For patients with mechanical heart valve prosthesis median TTR increased from 68% (25th-75th percentile 60%, 79%) during conventional treatment to 74% (25th-75th percentile 66%, 91%) in the PSM period (*p* < 0.001). INR variation (CV) was reduced from 21.0% during conventional treatment to 16.5% during PSM (*p* < 0.001) (Table 3). There was also a reduction in the percentage of subtherapeutic INR-values (≤ 1.5/2.0) (14% at conventional treatment vs. 5.0% at PSM, *p* < 0.001), but no difference in extreme high INR values (≥ 5.0) (0.6%) (Table 3).

**Table 3:** INR-measurements, time in therapeutic range, and extreme INR values during conventional treatment and patient self-management

	**Conventional treatment**	**Patient self-management**	***p*-value**
	**(*n* = 372, 93%)**	**(*n* = 285, 71%)**	
Total number of INR measurements	7881	6303	
Median interval between INR measurements, (25, 75% percentile), days	17.6 (12.3, 24.1)	7.3 (6.7, 8.6)	
Time in therapeutic range (TTR), median (25, 75% percentile), %	71.6 (60.0, 82.7)	78.6 (67.9, 91.7)	< 0.001*
INR, mean, SD	2.58 (0.59)	2.76 (0.47)	
INR variation (CV), median	21.0	16.5	< 0.001*
Extreme INR values			
INR values ≤ 2.0, %^a^	14	5.0	< 0.001**
INR values ≥ 5.0, %	0.6	0.6	

Results are based on INR measurements from the last 6–12 months of conventional treatment before training and the last INR 25 measurements in the self-management period before data collection

^*^Paired t-test

^**^Wilcoxon signed rank test

^a^ ≤ 1.5 for patients with mechanical ON-X prostheses in aortic valve position

### Mortality

Yearly mortality in the PSM period was 0.69% (*n* = 15). Among the reported cases, the predominant causes of death were attributed to other diseases (47%) and cancer (13%).

## Discussion

This quality assurance project, conducted within real-life clinical practice, found that the long-term effectiveness and safety of anticoagulant treatment during PSM is comparable to conventional treatment administered by GPs. The same patients were followed from the start of conventional treatment at the GP and during PSM, and no statistically significant difference was observed in the annual risk of hospitalizations due to severe clinical complications, such as severe bleeding or thrombosis. Nonetheless, a noticeable trend towards a reduced annual risk of hospitalization due to severe clinical complications was apparent during PSM compared to conventional GP treatment. This trend, however, did not attain statistical significance, likely due to the limited number of patients included in the study. Nevertheless, patients undergoing PSM demonstrated statistically significant higher TTR and achieved greater stability in anticoagulation, as evidenced by reduced variability in INR and fewer occurrences of extreme INR values, compared to conventional treatment.

### Risk of complications

Previous reported annual risk of serious bleeding/major hemorrhages and thromboembolic events during PSM in a real-world setting from other European countries are comparable with our results. Specifically, the risk of serious bleeding/major hemorrhages during PSM in real-world settings ranges from 0.5% to 2.3% [6, 7, 9, 11–13, 24] whereas our study reported a risk of 1.3%. Similarly, the annual risk for thromboembolic events during PSM varies between 0.50% and 1.6% in previous studies [6, 7, 9, 11–13, 24], compared to 0.67% in our study (Table 2). However, it's noteworthy that only one of these previous studies directly compared the outcomes of PSM with conventional treatment [13]. In that study, no significant differences were observed in the rates of serious bleeding/major hemorrhages and thromboembolic events between conventional treatment and PSM, even after 1 or 5 years of follow-up [13]. Similarly, a meta-analysis from RCTs concluded that there was no reduction in the risk of major hemorrhages when comparing self-testing or PSM with conventional treatment [2–4], consistent with the findings of our study. However, it's important to note that the meta-analysis also included patient self-monitoring alongside PSM, and the participants included in RCTs represent a highly selected group. As a result, direct comparisons with our study may be limited. Nevertheless, it's noteworthy that the proportion of spontaneous bleedings observed in our study was lower during the PSM period (26%) compared to the conventional treatment period (43%), potentially indicating a clinical benefit associated with more stable INR values.

The meta-analysis revealed that the risk ratio (RR) or hazard ratio (HR) for thromboembolic events was 0.58 and 0.51, respectively, during PSM compared to conventional treatment [2–4], which is slightly lower than our study's findings (RR = 0.65). Reported annual mortality during PSM in clinical practice have been documented at various intervals: 0.33% (after one year of follow-up), 1.08% (after 5 years of follow-up) [13], and 2.4% (after 3.3 years of follow-up) [6], compared to 0.69% in our study. Discrepancies in reported risks for complications and mortality may be attributed to variations in patient selection criteria, sample size, indications for VKA therapy, comorbidities, patient demographics (such as age), duration of observation, quality of training, and the quality of management of VKA therapy during the conventional treatment period.

As clinical events such as severe bleeding and thrombosis are relatively uncommon and primarily associated with TTR [25], the lack of a significant reduction in the yearly risk for thrombosis during PSM compared to conventional treatment in our study is likely attributable to the relatively small sample size [25].

### TTR, INR variation and extreme INR values

Comparison of TTR between conventional treatment and PSM in clinical practice has not been previously reported. However, the observed increase in TTR from conventional treatment to PSM aligns with findings from previous meta-analyses conducted in other countries [1, 3, 26]. Moreover, the TTR achieved during PSM in our study (78.6%) is consistent with values reported in previous studies conducted in real-world clinical settings, ranging from 71.5% [6] to 78.5% [11]. This is slightly higher than the TTR results reported in meta-analyses (ranging from 67.1% [25] and 74.8%) [3] and comparable to findings from a prior study conducted in Norway (TTR 78.1%) [19]. However, direct comparisons are challenging due to variations in study methodologies, as elucidated above. Nevertheless, previous research has demonstrated a correlation between TTR and clinical outcomes, as summarized by Samsa and Matchar [24]. Therefore, the significant increase in TTR from conventional treatment to PSM observed in our study suggests an improvement in the quality of anticoagulant therapy. Given that there were no differences in baseline characteristics between the “Patients that provided INR-results”-group (*n* = 285) and the entire cohort (“All patients”) (*n* = 400), it is reasonable to infer that the INR group represents the same population as the entire cohort.

The risk of thromboembolism and bleeding increase significantly at INR levels below 2.0 and above 5.0, respectively [26, 27]. Moreover, variability in INR measurements correlates with the incidence of thromboembolic events, bleeding events, and mortality [28]. Therefore, the reduction in INR values ≤ 2.0 and the decrease in INR variation observed during PSM compared with conventional treatment in our study further validate the safety and quality of PSM in our cohort. Although the percentages of extreme INR values are higher than those reported in previous studies [19, 20, 29], this is likely attributable to the real-world context, as the figures are calculated from a higher number of INR measurements and longer intervals between INR testing in remote areas.

### Patient selection

In this study, less than 5% of the patients selected for training by GPs in PSM at Nordland Hospital in Bodø were unable to complete the training program due to various reasons, including difficulties in adhering to weekly INR control and parallel INR measurements, cognitive impairment, or suspicion of abuse. Only a small subset of patients discontinued PSM due to a significant deviation exceeding 20% in repeated parallel INR measurements. Notably, two of these patients were diagnosed with triple-positive antiphospholipid syndrome (APS), highlighting the importance of identifying APS patients, as interactions between antiphospholipid antibodies and thromboplastin used in point-of-care (POC) INR testing may influence results [30]. In summary, the selection criteria for identifying suitable patients eligible for PSM in this study have proven to be successful. Nevertheless, some support from GPs also during PSM remains crucial for long-term safety, particularly for identifying cognitive dysfunction or the need for assistance in managing interactions with other medications or advanced surgical interventions.

### Strengths and limitations

This study represents the first longitudinal cohort study conducted in clinical practice settings in Norway, wherein the same patient cohort was followed from the initiation of warfarin therapy under conventional follow-up by a GP (median 2.45 years, 1958 patient-years) to long-term PSM) (4.99 years, 2242 patient-years). Comparable longitudinal studies conducted in clinical practice across other European countries have reported median follow-up durations ranging from 1 [11] to 4.3 years [7], and includes between 296 [11] and 2068 patients [31].

A unique aspect of this study is the acquisition of data directly from patient journals, ensuring that all events from the initiation of warfarin therapy were captured from original documents. The patient cohort represents approximately 20% of individuals trained in PSM across Norway, exhibiting diverse indications for warfarin therapy and spanning an age range from 15 to 85 years. A potential limitation is the uniform training of patients at a single hospital; however, it is important to note that the training program adheres to standardized protocols and is implemented across ten different hospitals nationwide. Consequently, the findings of this study are representative for patients undergoing PSM training by Noklus.

One limitation of this study is that patients were not treatment-naive to warfarin at the time of the intervention, which may introduce a learning effect that could impact the results. Consequently, the comparability between the two periods (conventional treatment and PSM) may be compromised. Ideally, a RCT design would have been employed to ensure that the observed outcomes were solely attributed to the training in PSM. However, RCTs involve highly selective patient, limiting the generalizability of the findings [32], and necessitating validation through real-world studies. Nevertheless, the results of previous meta-analyses of RCTs align with our findings [2–4]. Another limitation is that 13 patients received warfarin treatment for less than 3 months prior to the initiation of PSM training, potentially increasing their risk of adverse events during the early stages of warfarin therapy. However, as no severe clinical complications were observed in these patients during this period, this did not affect our results. Additionally, information regarding the pharmacogenetic genotypes of CYP2C9 and VKORC1 related to warfarin dosage and efficacy was not included, as these analyses are not routinely performed in Norway. Morever, the lack of adjustment for the increased risk of mortality associated with advancing age. As this study primarily serves as a quality assurance study, its primary objective is to ascertain the safety equivalence of PSM compared to conventional treatment. Furthermore, it is important to note the decline in the number of patients receiving warfarin treatment, from approximately 2% in 2012 to 0.6% in 2020 following the introduction of DOACs [33]. Consequently, GPs may possess reduced proficiency in managing these patients, potentially resulting in inferior quality conventional treatment compared to the outcomes observed in this study.

Previous estimates and economic models suggest that PSM becomes more cost-effective compared to conventional treatment after approximately ten years [5, 25]. Moreover, for our cohort, geographical conditions such as long travel distances to the GP office and limited accessibility may restrict the frequency of INR measurements during conventional treatment. Consequently, periods characterized by INR deviations from the therapeutic range may go undetected with conventional treatment, thereby increasing the risk of bleeding or thrombosis.

## Conclusions

This study, conducted within real-life clinical practice, found that PSM is as safe as conventional treatment administered by GPs for a highly selected group of patients. There was no statistically significant difference in the annual risk of hospitalization due to severe bleeding or thrombosis during long-term PSM of anticoagulation treatment, characterized by weekly INR measurements and concurrent dosage adjustments, compared to previous treatment administered by GPs (conventional treatment). However, there was an improvement in surrogate measures assessing the quality of VKA therapy, including an increased TTR, reduced variability in INR, and fewer extreme INR values during PSM.

## Data Availability

The datasets used during the current study are available from the corresponding author on reasonable request and if in accordance with current General Data Protection Regulation (GDPR) guidelines.

## Abbreviations

APS: Antiphospholipid syndrome
CRNM: Clinically relevant non-major
CT: Computed tomography
CV: Coefficient of variation
DOAC: Direct oral anticoagulants
GEE: General Estimated Equation
GP: General practitioner
HR: Hazard ratio
INR: International normalised ratio
MRI: Magnetic resonance imaging
Noklus: The Norwegian Organization of Quality Improvement of Laboratory Examination
NPR: Norwegian Patient Registry
OR: Odds ratio
PSM: Patient self-management
RCT: Randomised controlled trial
RR: Risk ratio
SAE: Severe adverse event
SD: Standard deviation
TRR: Time in therapeutic range
VKA: Vitamin K antagonist
